# Laparoscopic Management of Abdominal Echinococcosis: A Technical Report on Surgical Techniques and Outcomes

**DOI:** 10.7759/cureus.56130

**Published:** 2024-03-13

**Authors:** Iulian M Slavu, Octavian Munteanu, Valeriu Gheorghita, Florin Filipoiu, Bogdan Ursuț, Raluca Tulin, Ileana Dima, Iulian A Dogaru, Adrian Tulin

**Affiliations:** 1 Anatomy, “Carol Davila” University of Medicine and Pharmacy, Bucharest, ROU; 2 Infectious Disease, Agrippa Ionescu Emergency Clinical Hospital, Bucharest, ROU; 3 Anatomy and Embryology, “Carol Davila” University of Medicine and Pharmacy, Bucharest, ROU; 4 Endocrinology, Agrippa Ionescu Emergency Hospital, Bucharest, ROU; 5 General Surgery, Agrippa Ionescu Emergency Clinical Hospital, Bucharest, ROU; 6 Medicine, “Carol Davila” University of Medicine and Pharmacy, Bucharest, ROU; 7 General Surgery, Agrippa Ionescu Emergency Hospital, Bucharest, ROU

**Keywords:** medicine, parasitology, general surgery, laparoscopy, infectious

## Abstract

This technical report explores the efficacy and methodology of laparoscopic surgery for treating abdominal echinococcosis, a parasitic infection caused by Echinococcus granulosus. We highlight the zoonotic nature of the disease, which predominantly affects the liver and occasionally other organs, noting the challenge of its asymptomatic progression that complicates timely diagnosis and intervention. We detail our surgical technique using a standard laparoscopy kit to address abdominal hydatid cysts, emphasizing the critical importance of preventing cyst rupture and spillage to avoid recurrence and anaphylactic shock. We discuss considerations for opting for laparoscopy over open surgery, such as reduced postoperative morbidity, faster patient recovery, and lower costs, while also acknowledging limitations like restricted instrument movement and the absence of haptic feedback. We advocate hypertonic saline as the preferred scolicidal agent and strategies to minimize spillage and manage the residual cavity. In conclusion, we assert that laparoscopy offers a viable and effective treatment option for abdominal echinococcosis, emphasizing that optimizing outcomes for this benign condition hinges on careful patient selection and a conservative surgical approach.

## Introduction

Echinococcosis is a parasitic infection caused by the tapeworm Echinococcus granulosus (E. granulosus) [1]. It is a zoonotic disease, meaning it can be transmitted from animals to humans, with humans acting as accidental hosts in the parasite's lifecycle. The primary hosts are carnivores, typically dogs, in whose digestive tracts the parasite matures and releases eggs, known as a scolex, through feces onto grass [2]. Herbivores, such as sheep, ingest these eggs, continuing the lifecycle. In humans, the eggs hatch in the duodenum, an environment made conducive by its alkaline nature, and form larvae. These larvae travel through the portal system to primarily lodge in the liver (in 70% of cases) [3]. They can also bypass the liver, enter the systemic circulation, and develop in other organs, such as the lungs (this occurs in 25% of cases) [3]. Infected individuals often remain asymptomatic for extended periods, with cysts growing slowly and symptoms possibly emerging up to 15 years post-infection [4]. Symptoms arise from complications due to the cysts compressing surrounding structures or, in rare cases, cyst rupture [4]. Treatment aims to completely remove the parasite and prevent recurrence, utilizing various strategies based on the cyst's location, size, number, the patient's health, and the surgeon's expertise. Therapeutic options include medical treatment, surgery (either open or laparoscopic), and puncture aspiration instillation reabsorption [5]. Chemotherapy is recommended for one month post-treatment to reduce recurrence and anaphylaxis risks when the cyst is dissected or unroofed. Surgery is the preferred treatment for large cysts, such as Cystic Echinococcosis stage 3b (CE3b), providing an immediate cure. However, it is not recommended for small cysts (under 5 cm), uncomplicated or asymptomatic cysts, or when patients cannot tolerate general anesthesia [6,7]. This technical report details our experience treating abdominal hydatid cysts via laparoscopy, outlines our technique, and offers guidance for other surgeons.

## Technical report

Required instruments

We utilized a standard laparoscopy kit and its associated trolley. For good surgical outcomes, the kit should include at least two Maryland 5-mm forceps, a pair of scissors, a grasper, a hook laparoscopic instrument, bipolar forceps, and a percutaneous metal needle at least 5 mm in diameter and 15 cm in length (Veress needle). If possible, a 30-degree laparoscope should be used instead of a 0-degree one as it offers increased control of the image in tight locations of the operative field. The procedure will also require at least three trocars: two 10-mm and two 5-mm, a 37-cm sealing device, such as a Harmonic Scalpel (Ethicon, part of Johnson & Johnson, New Brunswick, NJ) or LigaSure (Valleylab, Boulder, CO), which, while not mandatory will assist in dissection and decrease the operative time. A 60-mm Guyon syringe will be required that will be connected to the Veress needle to infuse the scolicidal substance. As a scolicidal agent, 100 ml of hypertonic saline (HS) solution will suffice. The substance will be infused into the cyst via the Veress needle. To prevent contamination, a large Endo Bag (Medtronic, Dublin, Ireland) should be available to extract the cyst contents. The remaining cystic cavity should always be drained with an 18 Fr drainage silicone tube. An open surgical kit needs to be ready if conversion is required. Additionally, the anesthesia team should be informed of the potential of an anaphylaxis reaction upon the cyst's puncture and should have a resuscitation kit available.

Surgical intervention: steps and technique

**Figure 1:**
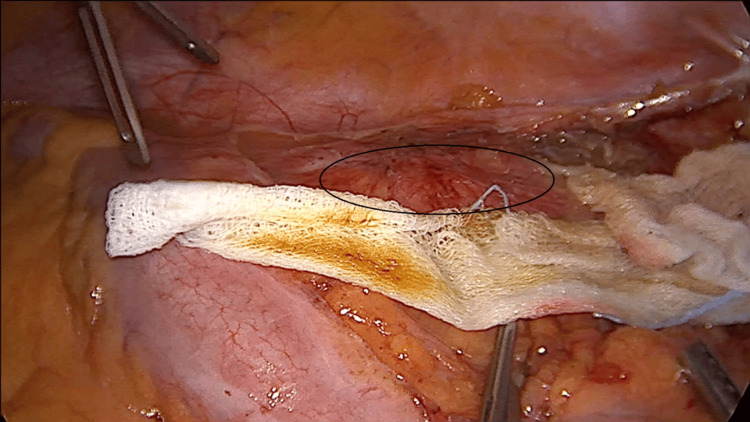
Laparoscopic view of the hydatid cyst and mesh placement around the cyst The cyst (black circle) is enclosed in sterile cloths soaked in hypertonic saline, serving as both a chemical and mechanical barrier to prevent spillage.

The location of the trocars in the abdominal wall is determined by the cyst's location, adhering to the triangulation principles as to avoid contact between the working trocars and the laparoscope. The 10-mm trocar is used for the camera, and another 10-mm working trocar is considered as a working trocar. This will also be used to extract the cyst contents via an Endo Bag. The final 5-mm trocar will also be used as a working trocar. After initial exploration of the abdomen, the cyst was identified. The adhesions are carefully resected so as to not put tension on the cyst’s capsule. Before inoculation of the scolicidal substance, the parasite is considered active and all steps should be taken to decrease the risk of rupture. During this step, we preferred using a sealing and cutting device to avoid tension on the adhesions during dissection. After gaining access to the cyst's dome, we isolated the working field with sterile cloths soaked in hypertonic saline, as shown in Figure 1.

**Figure 2:**
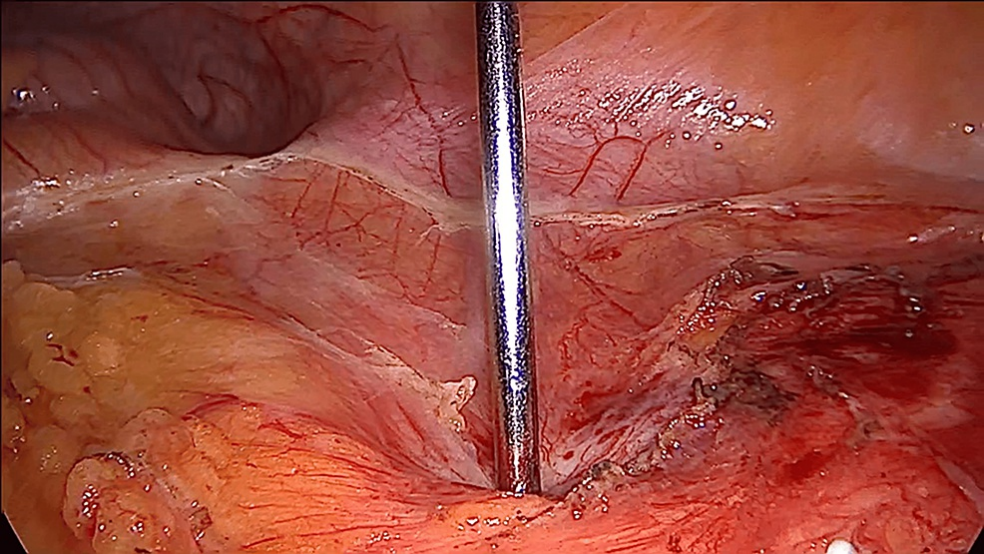
Laparoscopic view of the Veress needle penetrating the hydatid cyst Perforating the capsule, which is thick and fibrous, requires slight pressure on the needle.

These cloths acted as a chemical and mechanical barrier against intraperitoneal spillage. The next step required was to ensure parasite inactivation. An incision in the skin overlying the cyst was made through which the Veress needle was inserted into the cyst, ensuring it penetrated at least 3 to 4 cm, as depicted in Figure 2.

**Figure 3:**
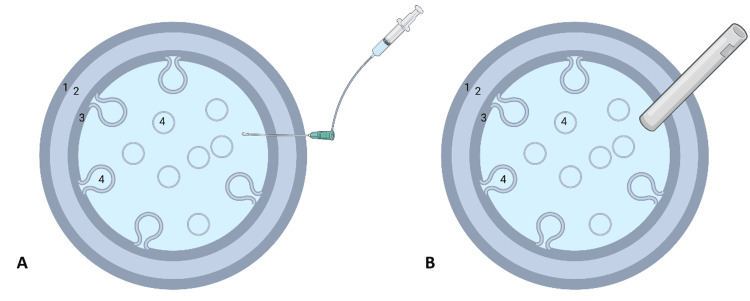
Schematic of hydatid cyst and parasite inactivation with hypertonic saline (A) Illustration shows the needle piercing the cyst, connected to a syringe filled with scolicidal agents via a catheter. (B) Post-inactivation, contents are extracted through a large cannula before unroofing. Key components labeled: 1 = Pericyst; 2 = Cyst; 3 = Proliger cuticular membrane; 4 = Daughter cyst (created using BioRender.com)

The needle should be inserted in the maximum fluctuance region of the cyst to ensure it enters the cyst’s cavity. This region can be felt by using two Maryland graspers, one in each hand, and moving them slowly over the dome of the cyst with minimal compression. During this maneuver, one can observe on the capsule the bulge made by the liquid in the cyst due to the compression with the graspers. It requires a bit of training but it is essential as the needle can be placed mistakenly in the wall and the cyst and the scolicidal substance cannot be administered. The resistance felt upon the cyst’s perforation with the needle will require a slight application of force. The risk of capsule tearing is minimal due to its surrounding fibrosis and thick walls. For this step, we use a Veress needle connected to a Guyon syringe as illustrated in Figures 3A, 3B.

**Figure 4:**
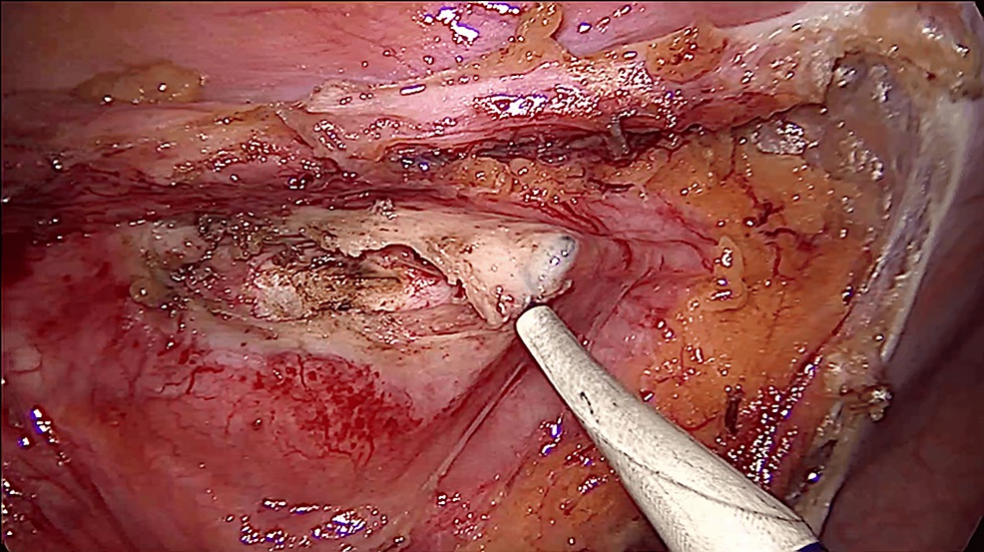
Laparoscopic view of the unroofing process The cyst's roof is penetrated using a hook. Despite the capsule's thickness, minimal bleeding occurs.

We slowly extracted approximately 50 mL of liquid, noting its quality-clear liquid indicates an active and alive parasite. In contrast, yellow or murky liquid suggested an infected cyst with a dead parasite. The scolicidal substance was then administered to the cyst. This consisted of 50 mL HS at 20% concentration. The substance has an active time of at least five minutes. Following this, we removed the needle, made a small incision in the cyst's roof with a Hook instrument, and introduced a suction device to evacuate the contents, using a standard surgical suction device, as shown in Figure 4.

If possible, as much of the cyst walls should be removed so as to decrease the relapse incidence. The dissection and resection will be aided by the cutting and sealing devices. The contents will need to be placed in an Endo Bag for extraction. The remaining cavity should always be drained as was the case of our patient with a silicone tube extracted through the abdominal wall in a sloping direction. Remember to note, count, and extract the sterile cloths used to isolate the parasite. These cloths should also be placed in an Endo Bag before extraction as to avoid further contamination. 

## Discussion

Surgery remains the cornerstone in the treatment of hydatid cysts, with a quality of evidence rated at level III and a recommendation strength of grade B, particularly for CE2 or CE3b cysts that are at risk of rupture or could compress adjacent organs [8]. Laparoscopic surgery offers the benefits of reduced postoperative morbidity, faster patient recovery, and decreased costs for the hospital [9]. The first laparoscopic operation for an abdominal hydatid cyst was reported in 1994 [10]. Initially, laparoscopy faced significant controversy due to concerns related to abdominal dissemination that can lead to a high recurrence rate and anaphylactic shock [10,11]. The complications were due to the necessity to puncture the cyst and inactivate the parasite before removal. During this process, hydatid liquid can leak in the peritoneal cavity beside the needle. With these negative aspects in mind related to laparoscopy, the advocates of open surgery mention that although this technique has a higher postoperative morbidity the procedure decreases the rate of relapse and spillage as the cyst can be dissected and removed in its entirety without the need to inactivate the parasite first. Noting these limitations, if the laparoscopic technique is to be adopted for a certain patient, careful consideration of the cyst's characteristics must be taken into account. The cyst should be of medium size (below 6 cm), uncomplicated status (without infection or perforation in adjacent organs), and should be unique in number. If the cyst is located in the liver, it should be placed in the anterior segments to ensure proper exposure for dissection and resection [12]. As previously mentioned, parasite spread in the peritoneal cavity during perforation for inactivation is one of the limitations of laparoscopy. There are different solutions to this and one of them which we employed with success is the isolation of the operative field with sterile ribbons which can be either soaked in HS or iodine solution [12]. These cloths act both as a mechanical and chemical barrier against spillage and will decrease the rate of recurrence [13]. After the isolation process, as demonstrated in Figure 1 the next step will require parasite inactivation with the help of a scolicidal substance which needs to be instilled in the cyst. This puncture can lead to intra-abdominal dissemination of the liquid. In our surgical department, if the patient tolerates it when opening or puncturing the cyst, we increase the abdominal pressure from the standard 10 to 12 mmHg to 13 to 14 mmHg after which we decrease it to the standard of 10 mmHg for the rest of the operative time. This tip has been mentioned by [14] and although there is no hard evidence to sustain it, from our point of view is logical and requires a minimum amount of effort. The scolicidal substance, we use is HS at a concentration of 5% as it is readily available, cheap, and efficient [15]. A short wait time of five minutes is required after inoculation for parasite destruction [16]. Other scolicidal agents like formalin, high-concentration ethanol (98%), cetrimide, hydrogen peroxide, or silver nitrate are cited in the literature and can be used [17]. Caution is advised when using high-concentration ethanol. Due to this high concentration, it is easily flammable. A spark generated by the electrocautery in the region where ethanol has been used can ignite a flame which in the case of ethanol is colorless and can lead to serious injuries, both to the patient and the surgical team [18]. After the five minutes have passed as demonstrated in Figure 4, the unroofing of the cyst can begin, and the contents can be extracted. For this step, one can use a standard Hook instrument or a sealing and cutting device. Once the unroofing is finished the contents of the cyst can be extracted with the help of a suction device (standard surgical aspiration device) will suffice. Other authors have proposed different solutions for this step: Palanivelul uses a large transparent cannula to perforate the cyst and extract the contents [19]. Saglam uses an adapted grinder and suction apparatus (from gynecologic surgery) to perforate the cyst and macerate the contents which are then removed [20]. An adapted liposuction device which consists of a long metal rod connected to a suction device has also been used to perforate the cyst and remove the contents with good results [21]. All of these methods have evolved to decrease the rate of spillage. After the contents have been removed the surrounding structure which is the cyst or pericyst needs to be dissected, resected, and removed either completely or partially. When possible, we consider that a conservative approach such as partial cystectomy or unroofing with external sloping drainage is sufficient as the disease is being and recurrence rates are low (2%-25%) [22]. Advocates exist for complete pericystectomy (removal of the entire cyst and surrounding fibrous tissue) as it is associated with lower rates of relapse [23]. This procedure entails important postoperative morbidity, and it is justifiable from our point of view only for small cysts under 5-6 cm [24]. Each procedure has its proponents and critics, with currently no gold standard. Treatment should be tailored to the individual patient. After resection, the walls of the cyst should be placed in an Endo Bag and extracted to prevent dissemination. The remaining cavity depending on location will need to be addressed. Various management strategies exist that include suture of the cavity walls, simple external drainage, or abandonment (leave it as it is) [25]. When possible, the remaining cavity should be drained using a silicone tube (16Fr-18Fr). This will decrease the incidence of local collections and facilitate in time the disappearance of the cavity [26]. The tube should be placed and extracted from the abdomen through a counter incision located below the level of the cyst to ensure sloping drainage of the liquid.

## Conclusions

Laparoscopy is a viable option for the treatment of single, uncomplicated abdominal hydatid cysts. The surgical intervention can be practiced with a standard laparoscopy kit. Preventing spillage to decrease relapse is essential necessitating the isolation of the operative field with sterile cloths soaked in HS or iodine.
